# PTPN13 Contributes to Ebola Virus-Induced Immune Dysregulation via Dephosphorylation of IRF3 and PI3K-p85

**DOI:** 10.3390/v18070729

**Published:** 2026-06-30

**Authors:** Abbey N. Warren, Maria Gonzalez-Orozco, Ivan Kuzmin, Sreeja Parameswaran, Ruben Soto Acosta, Birte Kalveram, Sarah van Tol, Adam Hage, Padmanava Behera, Yoatzin Peñaflor-Tellez, Maria I. Giraldo, William Russell, Matthew T. Weirauch, Alexander Freiberg, Alexander Bukreyev, Ricardo Rajsbaum

**Affiliations:** 1 Center for Virus-Host-Innate-Immunity, RBHS Institute for Infectious and Inflammatory Diseases, and Department of Medicine, New Jersey Medical School, Rutgers University, Newark, NJ 07103, USA; anw94@rutgers.edu (A.N.W.); pb682@njms.rutgers.edu (P.B.); yp482@njms.rutgers.edu (Y.P.-T.); 2 Department of Microbiology and Immunology, University of Texas Medical Branch, Galveston, TX 77555, USA; maridgon@utmb.edu (M.G.-O.); adam.hage@nih.gov (A.H.); migirald@utmb.edu (M.I.G.); anfreibe@utmb.edu (A.F.); abukreye@utmb.edu (A.B.); 3 Department of Pathology, University of Texas Medical Branch, Galveston, TX 77555, USA; ivkuzmin@utmb.edu (I.K.); rubensotoacosta@gmail.com (R.S.A.); bkkalver@utmb.edu (B.K.); 4 Galveston National Laboratory, University of Texas Medical Branch, Galveston, TX 77555, USA; 5 Center for Autoimmune Genomics and Etiology, Division of Allergy and Immunology, Cincinnati Children’s Hospital, Cincinnati, OH 45229, USA; sreeja.parameswaran@cchmc.org (S.P.); matthew.weirauch@cchmc.org (M.T.W.); 6 Department of Biochemistry and Molecular Biology, The University of Texas Medical Branch at Galveston, Galveston, TX 77555, USA; wirussel@utmb.edu; 7 Department of Pediatrics, University of Cincinnati College of Medicine, Cincinnati, OH 45229, USA

**Keywords:** Ebola virus (EBOV), innate immune response, type-I interferons (IFN-I), PI(3)K pathway, protein tyrosine phosphatase, phosphorylation

## Abstract

Ebola virus disease (EVD) is characterized by immune dysregulation and damaging hyperinflammation. We aimed to characterize the signaling pathways and regulatory mechanisms dysregulated during EVD. To avoid hyperinflammation, innate immune signaling is regulated by post-translational modifications (PTMs), including protein phosphorylation. Here, we show that the protein tyrosine phosphatase nonreceptor type 13 (PTPN13) negatively regulates Interferon (IFN)-β while also positively regulating the neutrophil chemoattractant CXCL1. Using vectors encoding IRF3 with mutations on phosphorylation sites, we identified Y292 on IRF3 as a PTPN13 target of dephosphorylation. Knockout of PTPN13 increased IRF3 phosphorylation and expression of IFNβ and IFN-stimulated genes (ISGs) following poly(I:C) stimulation. Intriguingly, depletion of PTPN13 during Ebola virus (EBOV) infection resulted in decreased IFNβ and ISG induction at later time points post-infection, which correlated with increased viral titers. We identified PTPN13-mediated dephosphorylation of the viral protein VP35 as one potential mechanism inhibiting virus replication. Additionally, the induction of inflammatory chemokines, including CXCL1, decreased in PTPN13 knockout cells late during EBOV infection. These effects could be explained by increased phosphorylation of the regulatory p85 subunit of PI3K. Dephosphorylation of p85 promotes its degradation, subsequently enhancing PI3K kinase activity and downstream signaling via AKT. Together, our study suggests that PTPN13 is involved in immune regulation and efficient antiviral responses by dephosphorylation of IRF3, EBOV-VP35 and PI3K-p85.

## 1. Introduction

Ebola virus disease (EVD) is an often lethal disease that can be accompanied by hemorrhagic fever and is caused by viruses of the genus *Orthoebolavirus* [1,2]. Most outbreaks of EVD have been caused by Ebola virus (EBOV), with the most recent outbreak in the Democratic Republic of Congo occurring from September 2025 through December 2025 [3]. EVD is characterized by an excessive inflammatory response due to immune system dysregulation, which ultimately causes detrimental inflammation and induces lymphopenia [4,5,6,7,8,9,10]. Heightened levels of inflammatory cytokines and chemokines have been shown to correlate with fatal outcomes of EVD [11]. Despite this correlation, the mechanisms behind EVD immune dysregulation that turn the response detrimental instead of protective remain unclear [5,11,12].

Innate immune responses to viral infection begin when pattern-recognition-receptors (PRRs) detect viral pathogen-associated molecular patterns (PAMPs), leading to the production of type-I Interferons (IFN-I), chemokines, and cytokines [13,14,15]. Viral RNA is recognized during replication by the RIG-I PRR, which triggers downstream signaling via the adaptor protein MAVS and the IKK-related kinases TBK1 and IKKε [16]. TBK1 and IKKε phosphorylate Interferon regulatory factor 3 (IRF3) at residue S386. In addition, it has also been proposed that c-Abl can phosphorylate IRF3 on residue Y292, further promoting phosphorylation on S386, causing IRF3 dimerization and activation [17,18,19]. Dimerization of IRF3 results in translocation to the nucleus where it couples with CBP/p300 to initiate IFNβ mRNA transcription [20]. IFN-I released from infected cells then signals via its receptor (IFNAR). The JAK1 and TYK2 kinases then phosphorylate the ISGF3 complex composed of transcription factors STAT1, STAT2 and IRF9, resulting in nuclear translocation and induction of multiple IFN-stimulated genes (ISGs), which are the actual effectors of the antiviral response [21,22,23,24,25].

During EBOV infection, the viral protein VP35, which acts as the viral co-factor of the polymerase [26], has been reported as a potent IFN-I inhibitor by impairing RIG-I’s ability to detect viral RNA [27]. Furthermore, VP35 has been shown to be phosphorylated by TBK1 and IKKε, effectively preventing IRF3’s phosphorylation through competition for binding to the kinases [28]. Because of VP35’s function as an IFN-I antagonist, VP35 has been associated with immune dysregulation and failure to control virus replication. Recombinant viruses encoding VP35 mutations that lack IFN-I antagonism replicate poorly in cells and in vivo [29,30,31,32,33].

An effective immune response requires a balance between clearing the virus and avoiding excessive inflammation and damage to the host. To this end, phosphorylation and dephosphorylation have been shown to be potent regulators of innate immune signaling by controlling enzyme activation and deactivation [34]. Phosphorylation can occur on three amino acids: serine (S), threonine (T), and tyrosine (Y). Tyrosine phosphorylation is carried out by tyrosine kinases while dephosphorylation of tyrosine sites is carried out by protein tyrosine phosphatases (PTPs) [35]. PTPs can also be classified as protein tyrosine phosphatases nonreceptor types (PTPNs), class I PTPs that are not membrane-bound [35]. Previous studies have revealed that PTPNs play crucial roles in immune regulation; for example, PTPN6 negatively regulates T-cell activation through ZAP70 and PTPN1/2 negatively regulate inflammation via JAK/STAT signaling [36,37,38]. Another member of this family, PTPN13, was previously reported to target and dephosphorylate Ikβα Y42 [39,40]. Dephosphorylation of IκBα at Y42 blocks dissociation of the IκBα subunit and prevents activation of NF-κB, blocking the induction of inflammatory genes [41]. PTPN13 has also been linked to positively regulating the AKT pathway through dephosphorylating p85β on residue Y655, leading to its ubiquitination and subsequent degradation [42]. Degradation of p85β releases p110, ultimately resulting in the downstream phosphorylation of AKT [42]. While PTPN13 has known immunoregulatory roles, how it regulates the immune response during EBOV infection has not been studied, and PTPN13 has not been previously linked to regulation of the IFN-I system.

Our aim for this study was to identify immune signaling pathways controlled by PTPNs that are dysregulated during EBOV infection. Here we report a novel regulatory role of PTPN13 in IFNβ induction and the neutrophil chemoattractant CXCL1. We demonstrate that PTPN13 negatively regulates IRF3 phosphorylation, leading to reduced IFNβ and ISG induction. PTPN13 promotes dephosphorylation of IRF3 on Y292, reducing subsequent phosphorylation on serine 396 (S396) and subsequent IFN-I induction. Additionally, we show that PTPN13 positively regulates the AKT pathway and induction of CXCL1. Furthermore, we show that, during infection, EBOV promotes an early and transient induction of PTPN13 mRNA and protein expression in WT cells. Reduced expression of PTPN13 at later time points correlates with an increased IFN-I response. PTPN13 knockout cells showed an early increase in IFN-I induction as compared to WT cells, which appears to control early viral replication. However, PTPN13 knockout cells display dysregulated gene expression at later time points post-infection, resulting in increased virus replication. Since fatal EBOV infections can correlate with an increase in CXCL1 levels [43,44], and dysregulated IFN responses [27,28], our study suggests that EBOV may target PTPN13 for immune dysregulation during EBOV infection and may contribute to EVD.

## 2. Materials and Methods

### 2.1. Cell Lines

HEK293T (CRL-11268), A549 (CCL-185), and THP-1 (TIB-202) were purchased from the ATCC (Manassas, VA, USA). HEK293T and A549 cell lines were maintained in Dulbecco’s Modified Eagle’s Medium (DMEM) (Corning, Corning, NY, USA) supplemented with 10% *v*/*v* fetal bovine serum (FBS) (Corning, Corning, NY, USA) and 1% *v*/*v* penicillin-streptomycin (Corning, Corning, NY, USA). THP-1 cells were maintained in ATCC-formulated RPMI-1640 medium supplemented with 10% *v*/*v* FBS, 1% *v*/*v* penicillin-streptomycin, and 0.05 mM β-mercaptoethanol.

BMDCs were prepared as described [45]. In brief, bone marrow cells were collected from C57BL/6J mouse femurs. Red blood cells were lysed. Remaining cells were incubated in RPMI with 20 ng/mL GM-CSF for 6 days. On the sixth day, CD11b+ CD11c+ cells were sorted at 98% purity (BD FACSAria Fusion–UTMB Flow Cytometry and Cell Sorting Core Lab) (BD, Franklin Lakes, NJ, USA).

### 2.2. Generation of PTPN13 Knockout Cell Lines

PTPN13 knockout cell lines were generated using the lentiCRISPR v2 plasmid (Addgene #52961) (Addgene, Watertown, MA, USA) produced by the Feng Zhang lab [46]. Single-guide RNA was designed using http://crispr.mit.edu:8079/ (accessed on 26 June 2026) and targeted the PDZ signaling domain of PTPN13. The sgRNA sequence was cloned into the lentiCRISPR v2 plasmid following the directions described [47]. To generate the knockout cell lines, 500,000 A549 cells per well and 400,000 293T cells per well were plated in 6-well plates. The cells were transfected with 1 µg of plasmid using Lipofectamine 3000 (Thermo Fisher Scientific, Waltham, MA, USA) or Mirus LT-1 (Mirus Bio, Madison, WI, USA), respectively, according to manufacturer recommendations. The A549 cells had their media replaced after 6 h. Twenty-four hours post-transfection, the media was replaced with DMEM containing 10% FBS and 1.5 µM puromycin for selection. Cells were grown in media containing puromycin until viable cell growth was detected. Once cells began growing, they were diluted and plated in 96-well plates to produce single clone colonies. Cells were expanded and then moved up to 6-well plates and finally a T75 flask once almost confluent. Knockout was verified by immunoblot.

### 2.3. Viruses

A549 and 293T cells were infected with EBOV strain Mayinga-76 originating from the CDC (CDC, Atlanta, GA, USA) and maintained at the University of Texas Medical Branch at Galveston. THP-1 cells were infected with the parental recombinant wildtype EBOV strain Mayinga-76 [48] along with a recombinant mutant EBOV called EBOV/VP35m with the following mutations introduced: F239A, R322A, and K319A [30]. Virus stocks were collected from passage 2 in Vero E6 cells following recombinant virus recovery. For infection of WT and PTPN13 KO cells, both viruses were used at MOI of 2 PFU/cell. BMDCs isolated from mice were infected using the mouse-adapted EBOV (maEBOV) at an MOI of 1 PFU/cell [49].

### 2.4. Replication and Transcription-Competent Virus-like Particles (trVLPs)

trVLP plasmids were a kindly provided by Dr. Mariano Garcia-Blanco (University of Virginia, Charlottesville, VA, USA). This system uses a minigenome (MG) and transcription-competent viral-like particle (VLP) that represent a lifecycle and expresses Renilla luciferase (Rluc). VP40, VP24, and GP are expressed from the MG, while NP, VP35, VP30, and L need to be transfected separately into passage 0 (p0) producing cells. The recovered supernatants (p1) contain VLPs that are competent for transcription, RNA replication, and production of infectious VLPs when NP/VP35/VP30/L are transfected again, and Rluc can be measured in p1-receiving cells.

trVLPs were produced and passaged as described [50]. In brief, trVLPs were prodpepuced in 293T cells, by transfecting p4cis-vRNA-RLuc (minigenome), pCAGGS-T7 polymerase, pCAGGS-L, pCAGGS-NP, pCAGGS-VP35, pCAGGS-VP30, and firefly luciferase, then collecting the supernatant after 72 h. Cells were prepared for infection with trVLPs by plating 50,000 293T cells per well in a 24-well plate, then transfecting pCAGGS-L, pCAGGS-NP, pCAGGS-VP35, pCAGGS-VP30, TIM1, firefly luciferase, and WT PTPN13 or PTPN13 DA. Twenty-four hours post-transfection, cells were infected with trVLPs for either 24 or 72 h. trVLP replication signal was measured using the Promega Dual-Luciferase system (Promega, Madison, WI, USA). In THP-1 cells, infections were performed with trVLPs without any additional transfection.

### 2.5. Plasmids

The GFP-IRF3, GFP-IRF3 5D, and reporter plasmids expressing firefly luciferase under the control of the IFNβ promoter were provided by Adolfo Garcia-Sastre (Icahn School of Medicine at Mount Sinai, New York, NY, USA). The plasmid carrying the Renilla luciferase gene (REN-Luc/pRL-TK) was purchased from Promega (Madison, WI, USA). The PTPN13 plasmid was purchased from Origene Technologies (Rockville, MD, USA). The PTPN13-DA mutant plasmid was generated by site-directed mutagenesis using the Agilent Technologies QuikChange XL Site-Directed Mutagenesis kit (Agilent Technologies, Santa Clara, CA, USA). The His-IRF3 Y292F mutant was purchased from VectorBuilder (Chicago, IL, USA).

### 2.6. PTPN13 Knockdown In Vitro Using siRNA

Transient knockdown of endogenous PTPN1, PTPN6, and PTPN13 in A549 cells, plated in 24-well plates at 30,000 cells per well, was achieved by transfection of ON-TARGETplus Non-targeting Control Pool (D-001810-10-05, Dharmacon, Lafayette, CO, USA), ON-TARGETplus PTPN1 SMARTpool siRNA (L-003529-00-0005, Dharmacon, Lafayette, CO, USA), ON-TARGETplus PTPN6 SMARTpool siRNA (L-009778-00-0005, Dharmacon, Lafayette, CO, USA), or ON-TARGETplus PTPN13 SMARTpool siRNA (L-008065-00-0005, Dharmacon, Lafayette, CO, USA) for a final concentration of 20 nM of siRNA for 48 h. Transfection was carried out by using Lipofectamine RNAiMAX (Invitrogen, Carlsbad, CA, USA) according to the manufacturer’s guidelines.

### 2.7. Stimulations

Poly(I:C) stimulation was carried out by transfecting cells with HMW Poly(I:C) at a concentration of 1 µg/mL using Lipofectamine 2000 (Invitrogen, Carlsbad, CA, USA). TNFα stimulation was carried out by serum starving cells in DMEM without FBS for 8 h, then replacing the media with DMEM containing 20 ng/mL of human TNFα (PeproTech, Cranbury, NJ, USA).

### 2.8. RNA Extraction and Quantitative Reverse-Transcription-PCR (qRT-PCR)

Cells were lysed in Trizol (Thermo Fisher Scientific, Waltham, MA, USA) or Tri-Reagent (Zymo Research, Irvine, CA, USA), then processed according to manufacturer’s instructions using the Direct-zol RNA kit (Zymo Research, Irvine, CA, USA). cDNA was synthesized through reverse transcription using the High-Capacity cDNA Reverse Transcription Kit (Applied Biosystems, Foster City, CA, USA) according to the manufacturer’s instructions. Real-time qPCR mixtures were made using iTaq Universal SYBR Green Supermix (Bio-Rad Laboratories, Hercules, CA, USA) and carried out in 384-well plates in triplicate using a CFX384 Touch Real-Time PCR Detection System (Bio-Rad). Gene expression was normalized to human 18S with the comparative CT method (ΔΔCT).

### 2.9. Immunoblots

Proteins were run on 7.5 Mini-PROTEAN and Criterion TGX SDS-PAGE gels (Bio-Rad Laboratories, Hercules, CA, USA) and then transferred to methanol-activated Immun-Blot PVDF membrane (Bio-Rad Laboratories, Hercules, CA, USA). The membrane was blocked in blocking buffer (5% powdered Carnation skim milk (Nestlé, Vevey, Switzerland) in 1X TBS-T) for 1 h. Primary antibodies were prepared in 1X TBS-T with 2% bovine serum albumin and 0.02% sodium azide to the designated dilution: anti-FLAG 1:2000 (Sigma F7425, Sigma-Aldrich, St. Louis, MO, USA), anti-GFP 1:1000 (Proteintech 50430-2-AP, Proteintech, Rosemont, IL, USA), anti-IRF3 1:1000 (Cell Signaling 10949S, Cell Signaling Technology, Danvers, MA, USA), anti-IRF3 S386 (Abcam ab76493, Abcam, Cambridge, UK), anti-TBK1 1:1000 (Novus NB100-56705, Novus Biologicals, Centennial, CO, USA), anti-TBK1 S172 (Abcam 3300-1, Abcam, Cambridge, UK), anti-PTPN13 1:1000 (Proteintech 25944-1-AP, Proteintech, Rosemont, IL, USA), anti-Actin 1:5000 (Sigma A1978- 100UL, Sigma-Aldrich, St. Louis, MO, USA), anti-Lamin A/C 1:1000 (Santa Cruz sc-376248, Santa Cruz Biotechnology, Dallas, TX, USA), anti-Tubulin 1:2000 (Sigma T8328-100UL, Sigma-Aldrich, St. Louis, MO, USA), anti-VP35 1:1000 (Kerafast Kf-Ab01229-2.0, Kerafast, Boston, MA, USA), anti-STAT1 1:1000 (Cell Signaling 14994S, Cell Signaling Technology, Danvers, MA, USA), anti-STAT1 Y701 1:1000 (Cell Signaling 7649L, Cell Signaling Technology, Danvers, MA, USA), anti-STAT2 1:1000 (Cell Signaling 72604S, Cell Signaling Technology, Danvers, MA, USA), anti-STAT2 Y690 1:1000 (Cell Signaling 88410S, Cell Signaling Technology, Danvers, MA, USA), anti-AKT 1:2000 (Cell Signaling 2920S, Cell Signaling Technology, Danvers, MA, USA), anti-AKT S473 1:2000 (Cell Signaling 4060S, Cell Signaling Technology, Danvers, MA, USA), and anti-p85β 1:1000 (R&D Systems MAB6777, R&D Systems, Minneapolis, MN, USA). Membranes were incubated in primary antibodies overnight on a rotating platform at 4 °C. The next day, the membranes were washed in 1X TBS-T three times before being incubated with secondary antibody prepared in blocking buffer at the following dilutions: HRP conjugated Goat anti-Rabbit IgG 1:10000 (Sigma NA934-1ML, Sigma-Aldrich, St. Louis, MO, USA) or HRP conjugated anti-Mouse IgG 1:10000 (Cytiva FENA931-1ML, Cytiva, Marlborough, MA, USA). After an hour of incubation in secondary antibody, the membrane was washed in 1X TBS-T three times and developed using Pierce ECL Western Blotting Substrate (Thermo Fisher Scientific, Waltham, MA, USA) or SuperSignal West Femto Maximum Sensitivity Substrate (Thermo Fisher Scientific, Waltham, MA, USA). Membranes were imaged using an Invitrogen iBright CL1500 Imager (Invitrogen, Waltham, MA, USA) or Bio-Rad ChemiDoc MP Imaging System (Bio-Rad Laboratories, Hercules, CA, USA).

### 2.10. Confocal Microscopy

A549 cells were seeded at a concentration of 30,000 cells per well onto coverslips placed in a 24-well plate. The cells had siRNA knockdown for PTPN13 performed as previously described for 48 h. After 48 h, the cells were transfected with Poly(I:C) as previously described for 4 h. After 24 h, the cells were washed once with DPBS and fixed for 10 min at room temperature with 4% (*v*/*v*) paraformaldehyde in DPBS. Cells were then washed three times with DPBS-T (DPBS with 0.1% (*v*/*v*) Tween-20) and permeabilized using 0.5% (*v*/*v*) IGEPAL CA-630 in DPBS for 10 min. Cells were washed three times using DPBS-T before being blocked in 1% BSA (*w*/*v*) in DPBS-T for 30 min. After blocking, cells were incubated in primary antibody (anti-PTPN13 1:200 (Proteintech 25944-1-AP, Proteintech, Rosemont, IL, USA) or anti-IRF3 1:400 (Cell Signaling 10949S, Cell Signaling Technology, Danvers, MA, USA) diluted in 1% BSA in DPBS-T overnight in a humidified chamber at 4 °C. The next day, cells were washed three times with DPBS-T before being incubated with secondary antibodies Alexa Fluor 488 (1:500 Thermo Scientific A-11001, Thermo Fisher Scientific, Waltham, MA, USA) and Alexa Fluor 555 (1:500 Thermo Scientific A-21428, Thermo Fisher Scientific, Waltham, MA, USA) and counterstained with DAPI (1:2000 Thermo Scientific D1306, Thermo Fisher Scientific, Waltham, MA, USA) diluted in blocking buffer for 1 h at room temperature. Cells were then washed three times with DPBS-T before the coverslips were mounted onto microscope slides using Fluor Save mounting media (Millipore, Burlington, MA, USA). Images were acquired using a Zeiss LSM 880 with Airyscan (Carl Zeiss, Oberkochen, Germany) at 63× magnification at the UTMB Optical Microscopy Core.

Images were quantified and analyzed with ImageJ software (version 1.54s) [51] by first separating the red (PTPN13), blue (DAPI), and green (IRF3) channels via micrograph. Then, the whole cell Region Of Interest (ROI) was generated manually in the green channel. ROI and was used to quantify the area and fluorescence to generate the IRF3 mean fluorescence. The nuclear ROI was generated by using a DAPI binary mask to calculate IRF3 nuclear fluorescence, and its value was subtracted from the whole cell ROI to calculate the cytoplasmic IRF3 mean fluorescence. Statistical analysis of cell fluorescence of each ROI or the nuclear/cytoplasmic ROI was performed and plotted with GraphPad Prism (version 10.6.1) (GraphPad Software, Boston, MA, USA).

### 2.11. Cellular Fractionation

Cytoplasmic and nuclear compartments were separated using the NE-PER nuclear and cytoplasmic extraction kit (Thermo Fisher Scientific, Waltham, MA, USA) following the manufacturer’s instructions.

### 2.12. Transfections and Co-Immunoprecipitation

HEK293T’s were plated in 6-well plates at 400,000 cells/well overnight, then transfected with 50 ng VP35, 250 ng WT PTPN13, 250 ng PTPN13-DA, 250 ng WT IRF3-GFP, or 250 ng GFP-IRF3 5D unless otherwise specified, using TransIT-LT1 (Mirus Bio, Madison, WI, USA) following the manufacturer’s recommendations. Twenty-four hours after transfection, 293T cells were lysed in RIPA buffer (50 mM Tris-HCl pH 8.0, 150 mM NaCl, 1% (*v*/*v*) IGEPAL CA-630, 0.5% (*w*/*v*) sodium deoxycholate, 0.1% (*v*/*v*) SDS) supplemented with protease inhibitor cocktail (Roche, Basel, Switzerland), 5 mM N-ethylmaleimide (Sigma-Aldrich, St. Louis, MO, USA), and 5 mM iodoacetamide (Sigma-Aldrich, St. Louis, MO, USA). Cell lysates were clarified through centrifugation at 15,000 rpm for 20 min at 4 °C. A total of 10% of the clarified lysate was added to 2x Laemmli sample buffer (Bio-Rad Laboratories, Hercules, CA, USA) containing BME and boiled for 10 min at 100 °C while the remaining lysate was mixed with 7.5 µL of anti-FLAG M2 EZview Red agarose beads (Sigma-Aldrich, St. Louis, MO, USA). Lysate was incubated with beads overnight on a rotating platform at 4 °C. The beads were then washed seven times with RIPA buffer and boiled in 2x Laemmli containing BME buffer for 10 min at 100 °C. His-tagged immunoprecipitation was carried out in a similar fashion, except RIPA buffer was supplemented with 50 mM imidazole, and proteins were bound to a Ni-NTA beads (QIAGEN, Hilden, Germany). Samples were then eluted using buffer consisting of pH 8.0 Tris HCl and 300 mM imidazole.

### 2.13. IFNβ Luciferase Reporter Assay

HEK293T cells were plated in 24-well plates at 50,000 cells/well and were transfected with 30 ng of IFNβ reporter plasmid together with 10 ng of Renilla luciferase, and 50 ng of GFP-IRF3, 25 ng of GFP-IRF3 5D, or 100 ng of His-IRF3 Y292F. Twenty-four hours after transfection, the cells were lysed and IFNβ promoter activity was measured using the Promega Dual-Luciferase Reporter Assay System (Promega, Madison, WI, USA) on a Cytation 7 Cell Imaging Multimode Reader (BioTek Instruments, Winooski, VT, USA) according to the manufacturer’s instructions. Values were normalized to Renilla luciferase.

### 2.14. Minigenome Reporter Assay

The monocistronic minigenome construct expressing the firefly luciferase gene was kindly provided by Dr. Bukreyev (The University of Texas Medical Branch, Galveston, TX, USA) [52]. 293T cells were plated (50,000 cells/well) onto 24-well plates in 5% FBS 1X DMEM for 24 h, and co-transfected with the following plasmids: EBOV minigenome (125 ng), pCEZ-VP30 (31.25 ng), pCEZ-NP (62.5 ng), pCEZ-L (500 ng), pC-T7 polymerase (125 ng), 100 ng of empty vector (pCAGGS) or pCAGGS-VP35, Renilla, and 100 ng of empty vector, WT PTPN13, or PTPN13 DA. Twenty-four hours after transfection, the cells were lysed and IFNβ promoter activity was measured using the Promega Dual-Luciferase Reporter Assay System (Promega, Madison, WI, USA) on a Cytation 7 Cell Imaging Multimode Reader (BioTek Instruments, Winooski, VT, USA) according to the manufacturer’s instructions. Values were normalized to Renilla luciferase.

### 2.15. Virus Infections and Plaque Assays

A549 and 293T cells were plated in DMEM supplemented with 10% FBS while THP-1 cells were plated in RPMI supplemented with 10% FBS. Virus inoculum was prepared in 2% FBS DMEM or RPMI. At the time of infection, the media was removed and 100 µL of inoculum was added. The cells were incubated with the inoculum for 1 h at 37 °C and rocked every 15 min. The cells were then washed three times with 1X DPBS to remove the inoculum and fresh 2% FBS DMEM or RPMI was added. At the indicated time points, supernatants and cells were collected for titration, immunoblot, and qPCR respectively.

Virus titers were determined by plaque assay on Vero E6 cells as previously described [52].

### 2.16. Mass Spectrometry of EBOV-Infected BMDCs

Samples lysed in Laemmli were treated with benzonase nuclease (Sigma-Aldrich, St. Louis, MO, USA; Cat. No. E8263) and incubated for 30 min at 37 °C. Protein concentration was determined using a BCA assay kit, and samples were normalized to 10 mg total protein for digestion using S-Trap Midi columns (ProtiFi, Fairport, NY, USA) and processed per manufacturer instructions at 37 °C overnight. Upon elution, peptide quantification was performed on 5 μL using the Pierce Fluorometric Peptide Assay (Thermo Fisher Scientific, Waltham, MA, USA; Cat. No. 23290). Aliquots of 1.45 mL (for enrichment) and 45 μL (for label-free quantification, LFQ) were dried in a SpeedVac concentrator.

The LFQ aliquot was resuspended to a final concentration of 555 ng/μL prior to addition of Biognosys iRT peptides (Biognosys AG, Schlieren, Switzerland) (1:10 ratio), resulting in a final concentration of 500 ng/μL. Peptides were resuspended in 1.6% ACN, 0.8% acetic acid, and 0.1% FA in LC–MS-grade water. Samples were centrifuged at 15,000× *g* for 10 min prior to injection of 1 μg onto a Thermo RSLC 3000 coupled to an Eclipse LC–MS system.

Peptide mixtures were analyzed by nanoflow liquid chromatography–tandem Mass Spectrometry (nanoLC–MS/MS) using an UltiMate 3000 RSLCnano System (Thermo Fisher Scientific, Waltham, MA, USA) coupled online to an Orbitrap Eclipse mass spectrometer (Thermo Fisher Scientific, Waltham, MA, USA) via a nanospray ion source. Peptides were directly injected onto an Aurora analytical column (75 µm × 25 cm, 1.6 µm particle size; IonOpticks, Fitzroy, VIC, Australia) at a flow rate of 300–350 nL/min. After equilibration of the column in 98% solvent A (0.1% formic acid in water) and 2% solvent B (0.1% formic acid in acetonitrile, ACN), 2 µL of sample (in solvent A) was injected at 300 nL/min. Peptides were separated using the following gradient: 2% B (0–5 min), 2–32% B (5–94 min), 32–42% B (94–97 min), 42–90% B (97–99 min), 90% B (99–100 min), 90–10% B (100–101 min), 10% B (101–102 min), 10–95% B (102–104 min), 95% B (104–105 min), 95–2% B (105–106 min), and 2% B for re-equilibration (106–120 min). Data were acquired in positive ion mode using a data-independent acquisition (DIA) method with staggered 8 Da isolation windows across *m*/*z* 400–900 and a 3 s cycle time. Full MS scans (*m*/*z* 350–2000) were acquired in the Orbitrap at 60,000 resolution (at *m*/*z* 400) in profile mode with a maximum injection time (IT) of 118 ms and an AGC target of 50,000. The S-lens RF level was set to 30. MS/MS spectra were generated by higher-energy collisional dissociation (HCD) and acquired in the Orbitrap at 15,000 resolution in centroid mode using a normalized collision energy (NCE) of 30%, an IT of 22 ms, and an AGC target of 400,000.

### 2.17. RNA-Seq Data Processing

WT and PTPN13 knockout cells were infected at a multiplicity of infection (MOI) 2 PFU/per cell with EBOV and RNA was isolated at 24 or 48 h post-infection. RNA was enriched for Poly(A) RNA from total RNA using NEBNext polyA module (NEB E7490, New England Biolabs, Ipswich, MA, USA). First- and second-strand synthesis, adaptor ligation, and amplification of the library were performed using the NEBNext UltraExpress RNA Library Preparation Kit as recommended by the manufacturer (New England Biolabs, Ipswich, MA, USA). Library quality was evaluated using an Agilent DNA-1000 chip (Agilent Technologies, Santa Clara, CA, USA) on an Agilent 2100 Bioanalyzer (Agilent Technologies, Santa Clara, CA, USA). Quantification of library DNA templates was performed using qRT-PCR and a known-size reference standard. Paired-end 75-base sequencing by synthesis was performed using Element Bioscience’s AVITI sequencer (Element Biosciences, San Diego, CA, USA) and Cloudbreak FreeStyle flowcell (Element Biosciences, San Diego, CA, USA) using protocols defined by the manufacturer.

RNA sequencing data were processed using the nfcore/rnaseq pipeline (version 3.11.1) [53,54]. Initial quality control of the raw sequencing data was performed using FastQC (version 0.11.9) (http://www.bioinformatics.babraham.ac.uk/projects/fastqc/, accessed on 26 June 2026). Low-quality bases and adapter sequences were then trimmed and filtered from the reads using Cutadapt (version 3.4) (DOI: https://doi.org/10.14806/ej.17.1.200) and Trim Galore (version 0.6.7) (DOI: https://doi.org/10.5281/zenodo.7598955), respectively. rRNA sequences were subsequently removed from the aligned data using SortMeRNA (version 4.3.4) [55] to eliminate potential contamination from non-target RNA species. STAR aligner (version 2.7.9a) [56] was used to align the trimmed reads to a custom reference genome of hg38 and Zaire ebolavirus (GenBank: GCA_000848505.1). The resulting alignments were sorted and indexed using SAMtools (version 1.16.1) [57]. To further evaluate the quality of the RNA sequencing data, several tools were employed, including RseqQC (version 3.0.1) [58], Qualimap (version 2.2.2-dev [59,60]), dupRadar (version 1.28.0) [61], and Preseq (version 3.1.1) [62]. These tools provide comprehensive assessments of various quality metrics, such as read distribution, GC content, duplication rates, and library complexity, ensuring reliable data for downstream analysis. For transcript quantification, Salmon (version 1.10.1) [63] was utilized to estimate the expression levels of transcripts. Finally, differential gene expression analysis was performed using DESeq2 (version 1.34.0) [64] in R 4.1.3 (R version 4.1.3 (R Foundation for Statistical Computing, Vienna, Austria). Genes were considered differentially expressed if they had a fold change of at least 1.5 and an adjusted *p*-value < 0.05. Sequencing data were submitted to GEO with accession ID: GSE324141.

### 2.18. Statistics and Reproducibility

Plaque assays and IFNβ luciferase assays were conducted in biological triplicates. The RNA-seq was done with biological duplicates. qPCR analysis was done in technical triplicates. Immunoblots and immunofluorescence images were done at least two times and representative experiments are shown. Analysis was done using Graphpad Prism (version 10.6.1) (GraphPad Software, Boston, MA, USA) and conducting a Two-way ANOVA with a *p*-value cutoff of 0.05.

## 3. Results

### 3.1. Members of the PTPN Family Show Different Expression Patterns During EBOV Infection

**Figure 1:**
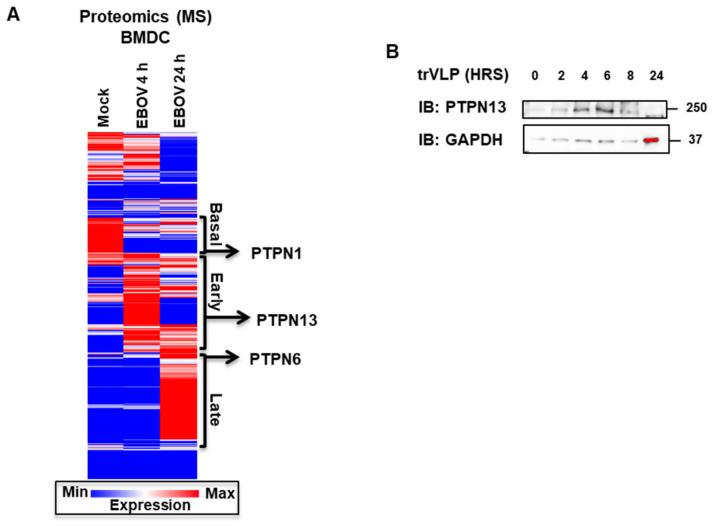
**PTPNs Display Differential protein Expression During EBOV Infection.** (**A**) BMDCs isolated from WT C57BL/6J mice were infected with maEBOV at an MOI of 1 PFU/cell for 4 or 24 h. Cells were lysed in Laemmli and analyzed for protein abundance by Mass Spectrometry. Three members of the PTPN family (PTPN1, PTPN6, and PTPN13) showed different temporal regulation patterns of protein abundance with PTPN1 upregulated at basal conditions, PTPN13 early, and PTPN6 late in infection. (**B**) THP-1 monocytes were infected with trVLPs and cells were lysed in Laemmli at the indicated time points for immunoblot analysis.

To identify potential post-translational modification (PTM) enzymes that could suggest dysregulation of the innate immune response during EBOV infection, Bone Marrow-Derived Dendritic Cells (BMDCs) isolated from WT C57BL/6J mice were infected with mouse-adapted EBOV (maEBOV) for 4 or 24 h. Protein was collected from mock and infected cells, then used for Mass Spectrometry analysis to measure changes in protein abundances over the course of EBOV infections (Figure 1A). Proteins were clustered in groups based on their abundance levels in basal (uninfected) conditions, or early (4 h) and late (24 h) during EBOV infection. We identified the PTPN family based on the differential expression pattern of three members during EBOV infection. PTPN1 showed its highest expression in uninfected cells, PTPN13 had its expression peak early during EBOV infection, and PTPN6 late during infection (Figure 1A). In line with the MS data, infection of human THP-1 monocytes with a transcription- and replication-competent virus-like particles (trVLPs) showed an early increase in PTPN13 protein expression, peaking at 6 h followed by a decrease at later time points post-treatment (Figure 1B), validating the MS data.

Based on previous reports of their involvement in immune regulation without connection to EBOV, we selected PTPN1, 6, and 13 for further investigation into their roles in immune dysregulation during EBOV infection.

### 3.2. PTPN13 Negatively Regulates IFNβ Production Through IRF3 Phosphorylation

**Figure 2:**
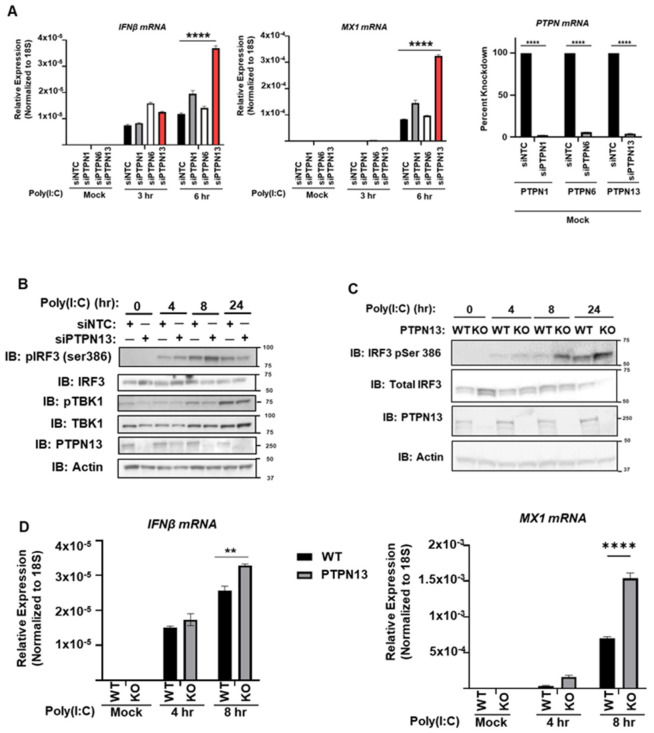
**PTPN13 Negatively Regulates IFNβ and ISG Induction.** (**A**–**D**) A549 cells were transfected with non-targeting control (NTC) or PTPN-targeting siRNA, followed by stimulation with Poly(I:C). At different time points, RNA was collected for qPCR analysis (**A**,**D**), or protein for immunoblot (IB) analysis (**B**,**C**). Data are presented as mean ± SEM. Statistical significance was determined using one-way ANOVA. ** *p* < 0.01; **** *p* < 0.0001.

To test the function of these PTPNs in immune regulation, we performed siRNA knockdown (KD) in A549 cells, followed by stimulation with the double-stranded RNA (dsRNA) mimic Poly(I:C), which induces IFN-I via RIG-I and MDA5 [65]. A significant increase in IFNβ mRNA was detected by qPCR in PTPN13 KD cells as compared to controls, at 6 h post-stimulation (Figure 2A, left panel). In line with this, expression of the ISG MX1 was also increased in PTPN13 KD cells (Figure 2A, right panel). Since PTPN13 was the only investigated protein to show significant differences upon knockdown and has not been previously associated with IFN-I responses, we focused our study on PTPN13.

In line with the results described above for IFNβ mRNA induction, IRF3 phosphorylation (on S386) was also increased in PTPN13 knockdown cells as compared to controls. In contrast, no changes were observed on TBK1 (Figure 2B), suggesting that PTPN13 acts downstream of TBK1/IKKε, possibly on IRF3, directly or indirectly. This increase in pIRF3 was reproduced in newly generated PTPN13 CRISPR-knockout (KO) A549 cell lines (Figure 2C). Poly(I:C) stimulation was repeated in these cell lines and a significant increase in IFNβ and ISGs mRNA was also detected following stimulation (Figure 2D), which correlated with an increase in IRF3 phosphorylation (Figure 2C).

**Figure 3:**
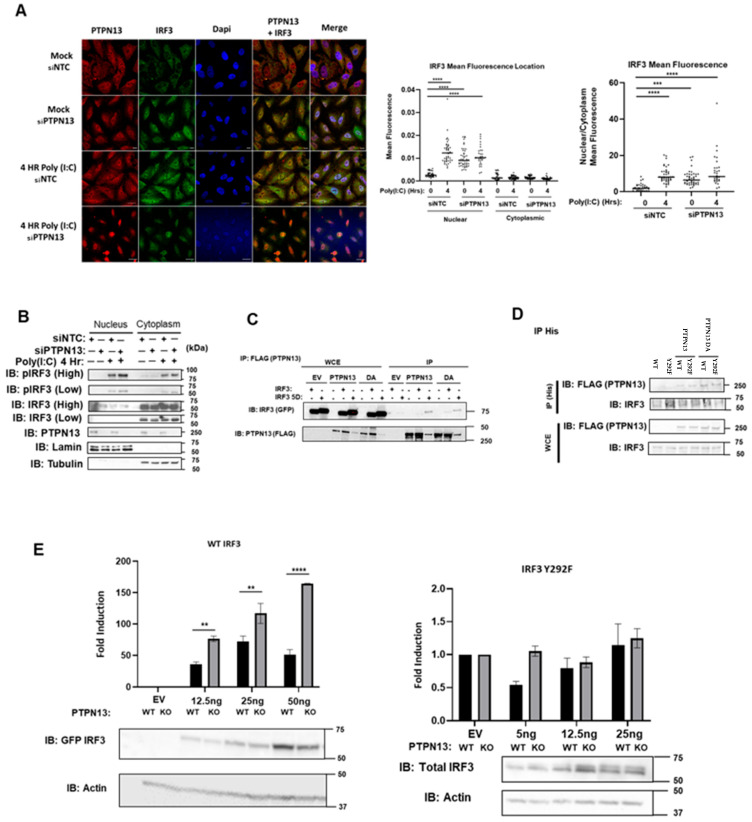
**PTPN13 Interacts with IRF3.** (**A**,**B**) A549 cells were transfected with non-targeting control (NTC) or PTPN13-targeting siRNA, followed by stimulation with Poly(I:C) treatment (4 h). IRF3 translocation to the nucleus was analyzed by confocal microscopy (**A**) or by IB after cell fractionation (**B**). (**C**) Whole cell extracts (WCE) from 293T cells transfected with plasmids for WT PTPN13 or PTPN13 DA and IRF3 WT or IRF3 5D were used for immunoprecipitation (IP) using anti-FLAG beads. (**D**) 293T cells were transfected with WT PTPN13 or PTPN13 DA and IRF3 WT or IRF3 Y292F, followed by IP using Nickel beads for His IP. (**E**) 293T WT or PTPN13 KO cells were transfected with WT or IRF3-Y292F mutant and the IFNβ luciferase reporter plasmids, followed by Dual-Luciferase assay. Data are presented as mean ± SEM. Statistical significance was determined using two-way ANOVA. ** *p* < 0.01; *** *p* < 0.001; **** *p* < 0.0001.

To test whether PTPN13 activity on IRF3 affects its nuclear translocation, we performed confocal microscopy and cell fractionation studies. We found that IRF3 translocation to the nucleus was increased in unstimulated PTPN13 KD cells by immunofluorescence and confocal microscopy (Figure 3A, quantification on the right panel). These effects were confirmed by fractionation assays, which showed enhanced phosphorylated IRF3 (pIRF3) nuclear translocation in PTPN13 KO cells, upon poly(I:C) stimulation (Figure 3B). These results suggest that PTPN13 reduces IRF3 phosphorylation, which also inhibits translocation (Figure 3A,B). To test whether PTPN13 may act directly by dephosphorylating IRF3, we generated a PTPN13 inactive mutant (PTPN13-DA) and we used an active phosphomimetic mutant of IRF3 (IRF3 5D). PTPN13-DA has Aspartic acid (D2359), located in the catalytic domain, mutated to an Alanine (A), rendering the phosphatase catalytically inactive but still capable of binding to and securing its substrates [36]. IRF3 5D is a constitutively active IRF3 mutant where sites S396, S398, S402, T404, and S405 were mutated to D to mimic phosphorylation [66]. Co-immunoprecipitation studies with overexpressed proteins showed that PTPN13 WT and IRF3 WT interacted weakly whereas PTPN13 DA showed increased binding affinity with IRF3 WT (Figure 3C). However, overexpression of IRF3 5D showed the greatest impact on binding between PTPN13 and IRF3, suggesting that PTPN13 interacts most efficiently with phosphorylated IRF3 (Figure 3C). Previously, it has been reported that IRF3 can be phosphorylated on Y292 by tyrosine kinase c-Abl [18]. We first tested whether an IRF3-Y292F mutant lacking this phosphorylation site would impact PTPN13’s binding with IRF3. Co-immunoprecipitation of WT PTPN13 or PTPN13 DA with His-tagged WT IRF3 or IRF3-Y292F showed that PTPN13 still co-immunoprecipitated with IRF3-Y292F; however, there was no clear decrease in binding between WT IRF3 and PTPN13 as compared to IRF3-Y292F and PTPN13 (Figure 3D). We still observe that PTPN13-DA binds more efficiently to both the WT and IRF3-Y292F mutant, consistent with its trapping function. This suggests that phosphorylation on Y292 (or a Y-to-F mutation) does not affect PTPN13’s binding with IRF3. Next, to test whether PTPN13 activity is connected to Y292, we performed an IFNβ promoter luciferase assay by overexpressing WT IRF3, IRF3 5D, or IRF3 Y292F and measuring luciferase activity in WT or PTPN13 KO 293T cells (Figure 3E). The results showed that, while IRF3 WT overexpression leads to increased IFNβ reporter activity in KO cells as compared to WT cells, in a dose-dependent manner, the IRF3 Y292F mutant is inactive. This suggests that the presence of an intact Y292 residue on IRF3 is required for optimal IFN induction.

### 3.3. PTPN13 Knockout Cells Display a Dysregulated ISG Response During EBOV Infection

**Figure 4:**
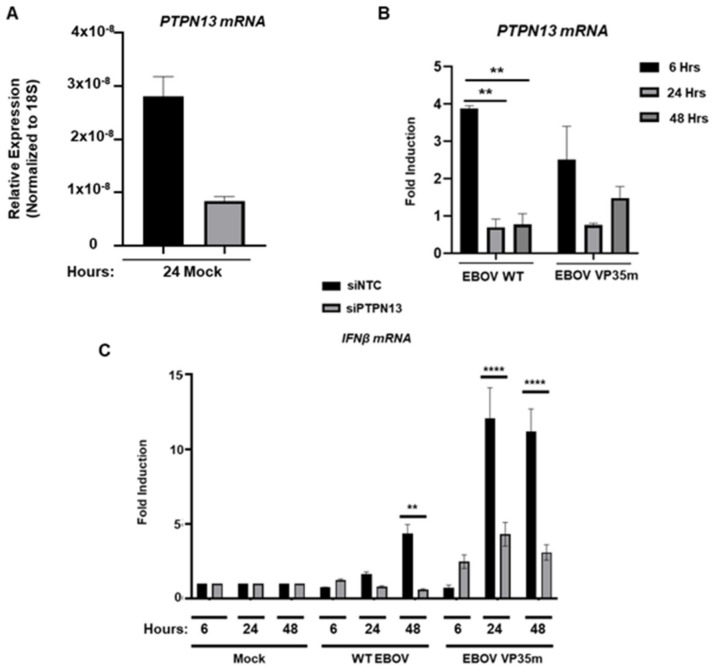
**PTPN13 KD in THP-1 Cells shows an Early Increase in IFN.** (**A**–**C**) THP-1 cells were treated with siNTC or siPTPN13 for KD, then infected with WT or EBOV/VP35m EBOV at an MOI of 5 for 6, 24, and 48 h; cells were lysed for RNA extraction and qPCR analysis. (**A**) Validation of siRNA KD of PTPN13 by qPCR in non-infected THP-1 cells. PTPN13 (**B**) and IFNβ (**C**) mRNA expression by qPCR. Data are presented as mean ± SEM. Statistical significance was determined using two-way ANOVA. ** *p* < 0.01; **** *p* < 0.0001.

We then asked whether PTPN13 may play a role during EBOV infection by inhibiting IFN-I. First, we performed knockdown of PTPN13 in the monocytic THP-1 cell line, which is a target for EBOV infection [67]. The efficiency of the knockdown was validated in mock samples by qPCR (Figure 4A). For comparison purposes, we also used a recombinant mutant virus of EBOV encoding mutations on VP35 [30]. Consistent with our data described above, in the control cells, PTPN13 mRNA was quickly upregulated at 6 h post-infection, followed by downregulation at later time points during WT EBOV infection (Figure 4B). Importantly, a recombinant EBOV VP35 mutant virus (VP35m) that lacks IFN-I antagonism [30] showed a similar effect on PTPN13 expression (Figure 4B), suggesting that IFN-I is not likely to be the reason for changes in PTPN13 expression. Although not statistically significant, IFNβ mRNA expression was slightly increased in PTPN13 knockdown cells infected with WT virus early, at 6 h post-infection, and this increase was more pronounced with the EBOV/VP35m virus (Figure 4C). Surprisingly, at 24 and 48 h, the PTPN13 knockdown cells showed less IFNβ production when compared with the control cells (Figure 4C). As expected, the EBOV/VP35m virus induced higher levels of IFNβ as compared to WT-EBOV in siControl-treated cells. These results suggest that, during EBOV infection, the loss of PTPN13 results in an indirect inhibitory effect on IFNβ induction occurring at later time points post-infection.

**Figure 5:**
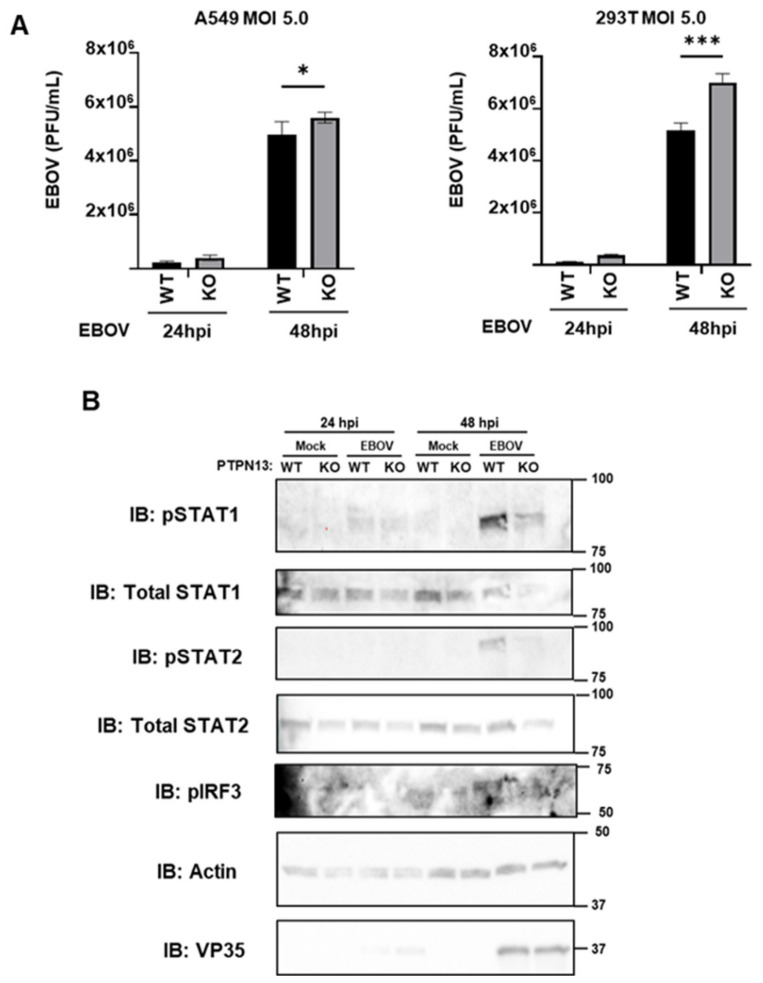
**PTPN13 KO Cells Display Increased EBOV Replication Correlating with Decreased ISGs.** (**A**,**B**) WT and PTPN13 KO A549 and 293T cells were infected with EBOV at an MOI of 2 for the indicated time points. Data are presented as mean ± SEM. Statistical significance was determined using two-way ANOVA. * *p* < 0.05; *** *p* < 0.001. (**A**) Supernatants were collected to determine viral titers by plaque assay. (**B**) PTPN13 KO and WT A549 cells were lysed in Laemmli to collect protein.

To further confirm these data, and test different cell types, we repeated the EBOV infection experiment using our newly generated PTPN13 A549 and 293T knockout cell lines. We found a slight but statistically significant increase in EBOV titers in both A549 and 293T PTPN13 knockout cells as compared to their respective controls (Figure 5A). In line with these data, immunoblot analysis showed a decrease in IRF3, STAT1 and STAT2 phosphorylation in PTPN13 knockout cells as compared to WT cells (Figure 5B). A decrease in STAT1 and STAT2 signaling in KO cells correlates with a decrease in ISG production, which could explain the increase in EBOV titers seen in KO cells at 48 h post-infection.

**Figure 6:**
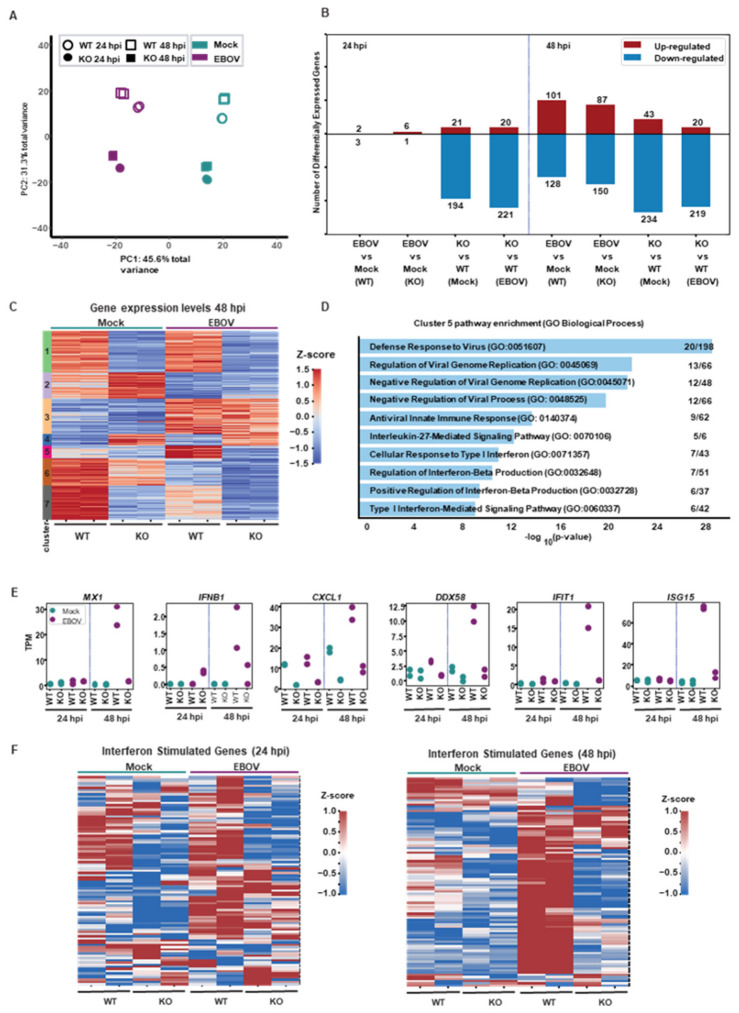
**RNA-seq analysis of PTPN13 KO cells in the context of EBOV infection.** WT and PTPN13 KO A549 cells were infected with EBOV at an MOI of 2. Following infection, samples were lysed in Trizol for RNA extraction and qPCR analysis. (**A**) Principle component analysis indicating the relationship between each experimental condition. (**B**) The number of differentially expressed genes in each pairwise comparison of conditions. Genes were considered differentially expressed if they had a fold change of at least 1.5 and an adjusted *p*-value < 0.05. (**C**) Heatmap displaying expression levels of differentially expressed genes across conditions 48 hpi. Clusters were identified using k-means clustering with k = 7. Cluster 5 includes genes that are highly expressed only in WT cells with EBOV infection. (**D**) Pathway enrichment analysis of Cluster 5 genes using GO Biological Process. Numbers at the left right indicate the number of genes in Cluster 5 that are also contained in the indicated GO Biological Process category, along with the size of that category. (**E**) Gene expression levels across conditions for select Cluster 5 genes. Y-axis values indicate RNA-seq-derived transcripts per million (TPM) values. (**F**) Heatmaps showing gene expression levels of Interferon Stimulated Genes (ISGs) at 24 hpi (**left**) and 48 hpi (**right**).

We next collected RNA from the WT and KO cells infected with EBOV to observe global changes in gene expression, via bulk RNA sequencing (RNAseq). Principle component analysis (PCA) showed distinct separation between the WT and KO samples with the replicates tightly clustered together (Figure 6A). We found that only a small number of genes were differentially regulated by EBOV infection at 24 h in either WT or KO cells, suggesting that EBOV early during infection does not trigger substantial changes in immune pathways. This is consistent with strong inhibition by VP35. However, at 48 h post-infection, when the virus reaches higher titers, a larger subset of genes are differentially expressed. Interestingly, a larger number of genes are downregulated in PTPN13 KO cells as compared to WT cells (Figure 6B,C). Genes were clustered based on their expression patterns (Figure 6C) and gene ontology (GO) analysis (Figure 6D). A set of genes (Cluster 5) which contained genes that were highly upregulated in the WT cells but not in the PTPN13 KO cells during EBOV infection (Figure 6C). GO analysis revealed that cluster 5 was highly enriched for genes in response to viral infection, including defense response to viruses and regulation of viral genome replication (Figure 6D). This cluster included genes such as *IFNB1*, *MX1*, *CXCL1*, *IFITM1*, *ISG15*, and *DDX58* (Figure 6E).

In line with our THP-1 experiment, IFNβ mRNA was slightly increased early during EBOV infection in KO cells (Figure 6E, Panel 2, 24 h); however, by 48 h the phenotype reversed and WT cells showed increased IFNβ mRNA expression as compared to PTPN13 KO cells (Figure 6E, Panel 2, 48 h). Consistent with this, decreased ISGs (MX1, ISG15, and IFITM1) levels were observed in PTPN13 KO cells as compared to WT cells (Figure 6E, Panels 1, 5, and 6). This pattern was also observed for the inflammatory cytokine and neutrophil chemoattractant CXCL1 (Figure 6E, Panel 3). These effects were consistent for genes in Cluster 5 across a large number of IFN-responsive genes, especially at 48 h post-infection (Figure 6F). Together, these data suggest that, while PTPN13 KO cells show an early spike in IFN-I, presumably due to increased IRF3 phosphorylation, depletion of PTPN13 results in a late indirect dysregulated IFN response.

### 3.4. VP35 Interacts with PTPN13

**Figure 7:**
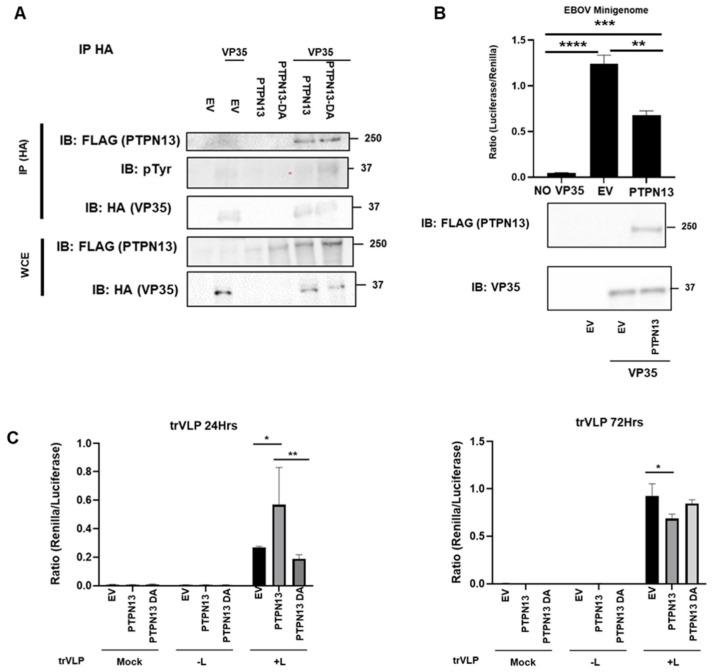
**PTPN13 Interacts with VP35**. (**A**) WCE from 293T cells transfected with VP35 and WT PTPN13 or PTPN13 DA were used for IP using anti-HA beads, followed by immunoblot. Tyrosine phosphorylation was analyzed with a specific antibody in the enriched VP35 fractions. (**B**) EBOV minigenome luciferase assay was performed from 293T cells transfected with empty vector (EV) or WT PTPN13, in the presence or absence of VP35. (**C**) trVLP assay. 293T cells were transfected with trVLP plasmids as described in Section 2, together with WT PTPN13 or PTPN13 DA. At 24 h post-transfection, cells were infected with trVLP and cells were lysed at the indicated time points for luciferase assay. A control without L overexpression was used as a negative control, indicating L is necessary for transcription/replication. Data are presented as mean ± SEM. Statistical significance was determined using two-way ANOVA. * *p* < 0.05; ** *p* < 0.01; *** *p* < 0.001; **** *p* < 0.0001.

VP35 is known to be phosphorylated, and this enhances virus replication [68]. To test whether PTPN13 could potentially inhibit the function of VP35 as co-factor of the viral polymerase via dephosphorylation of VP35, we examined whether ectopically expressed PTPN13 can interact with and dephosphorylate VP35, by co-immunoprecipitation (Figure 7A). We found that both WT PTPN13 and the PTPN13-DA mutant co-immunoprecipitated with VP35 (Figure 7A, top panel). Furthermore, a reduction in tyrosine phosphorylation on VP35 was observed in the presence of WT PTPN13 but not the PTPN13-DA mutant, suggesting that PTPN13 can promote dephosphorylation of VP35. These effects correlated with reduced VP35-mediated polymerase activity in a minigenome assay (Figure 7B). This minigenome experiment validates the PTPN13 phenotype observed at late time points post-infection in PTPN13 KO cells. Since this minigenome assay represents VP35 activity as a co-factor of the polymerase independent of its effects on IFN-I, it suggests that PTPN13 can dampen virus replication. In support of this, infection with a transcription-competent viral-like particle (trVLP) expressing Renilla luciferase (Rluc), which is representative of the viral lifecycle [50] and sensitive to the effects of IFN-I [69], confirmed PTPN13’s impact on EBOV replication (Figure 7C). In line with the effects observed with infectious EBOV, increased trVLP signal was detected at 24 h p.i. in PTPN13 but not PTPN13-DA overexpressing cells (Figure 7C, left panel). As with the infectious virus, the phenotype was reversed at later time points post-infection, with decreased trVLP signal in WT PTPN13 overexpressing cells (Figure 7C, right panel). The early increase in trVLP signal could be explained by PTPN13 overexpression dampening IRF3 phosphorylation and IFN-I induction, whereas the decrease at 72 h could be explained by PTPN13 dephosphorylating VP35 and/or other indirect PTPN13 effects on cytokine signaling. These overexpression experiments confirm that PTPN13 expression is beneficial for the virus early during viral replication, but not at later time points in the replication cycle.

Overall, this suggest that VP35 dephosphorylation by PTPN13 inhibits VP35 activity as the co-factor of the viral polymerase, reducing virus replication.

### 3.5. PTPN13 Positively Regulates CXCL1 Production by Promoting p85β Degradation

**Figure 8:**
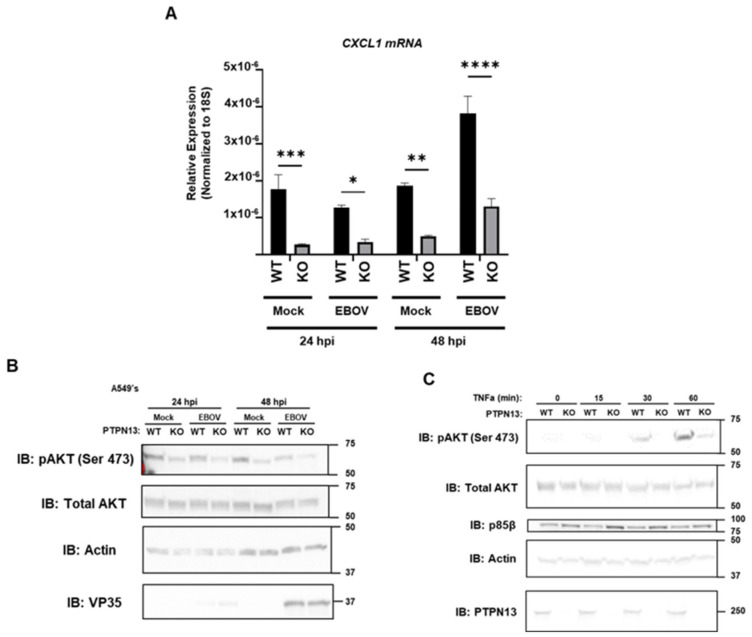
**PTPN13 Positively Regulates AKT Phosphorylation.** WT and PTPN13 KO A549 and 293T cells were infected with EBOV at an MOI of 2 for 24 or 48 h. Cells were lysed in Trizol to collect RNA for qPCR (**A**), or Laemmli to collect protein for immunoblot (**B**). (**C**) WT and PTPN13 KO A549 cells were serum-starved for 8 h, then stimulated for 15, 30, and 60 min using 20 ng/mL. Data are presented as mean ± SEM. Statistical significance was determined using two-way ANOVA. * *p* < 0.05; ** *p* < 0.01; *** *p* < 0.001; **** *p* < 0.0001.

Gene ontology analysis from the RNAseq experiment showed a group of cytokines and chemokines, with differentially regulated expression in EBOV-infected PTPN13 KO cells. Downregulated chemokines include those of the CXCL family, with members CXCL1, CXCL5, CXCL8, and CXCL10 downregulated at 48 h post-infection in the PTPN13 knockout cells. Importantly, expression of CXCL1, a neutrophil chemoattractant and previously proposed to be correlated with EVD [43,44], was significantly decreased in PTPN13 knockout cells as compared to WT cell, in the RNAseq and confirmed by qPCR (Figure 6E panel 3 and Figure 8A). In line with this, a decrease in AKT phosphorylation in PTPN13 knockout cells was observed by immunoblot (Figure 8B).

Since TNFα is produced during infection, and is also known to induce CXCL1 via activation of the PI3K-AKT pathway [70], we examined the role of PTPN13 in regulating TNF-mediated AKT phosphorylation. Upon TNFα stimulation of serum-starved cells, a reduced phosphorylation of AKT was observed in PTPN13 KO cells. (Figure 8B). Consistent with previous reports that PTPN13 regulates p85β phosphorylation and protein degradation [42], PTPN13 KO cells showed increased levels of p85β protein as compared to WT cells (Figure 8C). This suggests that PTPN13 positively regulates CXCL1 via activation of PI3K by dephosphorylating p85β, promoting its degradation, and subsequently releasing the catalytic p110 subunit for downstream signaling.

## 4. Discussion

Infection with Ebola virus causes Ebola virus disease, a severe and often lethal disease that can be accompanied by hemorrhagic fever. EVD is characterized by hyperinflammation resulting from a cytokine storm induced by EBOV-mediated immune dysregulation. Despite some features of hyperinflammation and immune dysregulation being linked to fatal outcomes in EVD, the regulatory mechanisms that are targeted during infection are not well-characterized [11]. Further, the molecular mechanisms that regulate the immune response and are disrupted during infection have not been fully elucidated. Here we found that the phosphatase PTPN13 interacts with phosphorylated IRF3, triggering dephosphorylation at Y292 to ultimately reduce IFN-I induction. We also show that PTPN13 was quickly upregulated during EBOV infection, then downregulated at later time points post-infection. PTPN13’s upregulation early during EBOV infection was validated in infected THP-1 monocytes, indicating that EBOV infection regulates PTPN13 expression levels in different cell types and in both mouse and human systems. Upregulation of PTPN13 occurs in EBOV infection as early as 2 h post-infection in THP-1 cells. This very early increase in PTPN13 expression suggests that virus infection (a viral protein), or an early cell intrinsic signaling pathway, and not a secreted cytokine, is responsible for PTPN13 upregulation. A potential explanation is that EBOV induces PTPN13 expression early to dampen the IFN-I antiviral response, giving the virus an advantage. PTPN13 has not been previously reported to play a role in the immune response to EBOV infection; therefore, we present here evidence that PTPN13 can affect EBOV replication via IFN-I as well as targeting VP35. It is important to note that, while PTPN13 expression levels may not necessarily correlate with phosphatase activity, the reduced expression at later time points post-infection suggests a correlation between reduced PTPN13 activity and increasing levels of IFN-I induction in WT cells. This could be a mechanism used by the host to counter the inhibitory effects of VP35 when present at higher levels later during infection. Since PTPN13 also appears to have direct antiviral activity by dephosphorylating VP35 and reducing polymerase activity, the changes in PTPN13 expression could benefit the host or the virus, depending on the time points. An additional possibility is that, in the absence of PTPN13 in KO cells, the function of EBOV VP24 in antagonizing the IFN-I signaling pathway is enhanced, further reducing ISG induction. It is known that VP24 binds to karyophin alpha in the same domain as phosphorylated STAT1, preventing its nuclear translocation and ISG induction [71]. Although so far, VP24 has not been reported to undergo phosphorylation, this could alter its ability to bind karyophin. One future direction could be to examine whether PTPN13 affects VP24 antagonist activity.

To determine PTPN13’s role in immune regulation but excluding any effects of virus replication, PTPN13 knockdown cells were transfected with a double-stranded RNA mimic poly(I:C). This stimulation serves as a mimic of virus infection, but excludes the effects of virus replication as well as excluding the antagonistic effects of VP35. These experiments helped dissect and demonstrate the role of PTPN13 in the negative regulation of IRF3 phosphorylation and IFN-I induction, which are not evident in the presence of the virus, but do show an early effect in the IFN-I response. PTPN13 knockdown resulted in an increase in IFNβ production and a corresponding increase in ISG induction. Probing the IFNβ induction pathway revealed an increase in IRF3 phosphorylation at S386, indicating an increase in IRF3 activity. TBK1 did not show any changes in phosphorylation status that correspond to changes in kinase activity. Enhancement of IRF3 activity but not TBK1 or IKKε implied that PTPN13 negatively regulated IFNβ production directly or indirectly at the level of IRF3. Immunoprecipitation of PTPN13 with IRF3 supports the hypothesis that PTPN13 may directly interact with IRF3 or, alternatively, occur together in a complex. PTPN13 showed increased binding efficiency with IRF3 when overexpressed with the IRF3 5D mutant, implying that PTPN13 requires the presence of phosphorylation on IRF3 for efficient interaction between these proteins to occur. Of note is that PTPN13 is a tyrosine-specific phosphatase, while IRF3’s main phosphorylation sites that control its activity are serine or threonine. However, it has been previously reported that IRF3 can be phosphorylated by c-Abl at site Y292, which promotes phosphorylation at site S386 [19]. One limitation is that IRF3 has no commercially available antibodies specifically targeting phosphorylated Y292, so we could not immunoblot for specific phosphorylation changes at this site. Instead, we made use of an IRF3 Y292F mutant. This IRF3 mutant appears mostly inactive in the absence of additional stimulus, suggesting that this phosphorylation is important for subsequent serine phosphorylation on IRF3. These data could imply that PTPN13 dephosphorylates IRF3 at Y292, subsequently, reducing IRF3’s phosphorylation at S386 and IFNβ production. At this time, we could not rule out whether PTPN13 may indirectly affect IRF3 phosphorylation at S386. Future studies using Mass Spectrometry analysis will help confirm whether the effects of PTPN13 on IRF3 dephosphorylation are specific to the Y292 residue.

We expected that depletion of PTPN13 would promote clearance of viral infection due to increased IFNβ production. Surprisingly, PTPN13 knockout cells showed only a marginal increase in IFN induction, which was downregulated at later time points post-infection, ultimately correlating with an increase in virus replication. Overexpression of PTPN13 confirmed a time-specific effect of virus replication. One possibility is that EBOV infection promotes the expression of a cellular factor in PTPN13 KO cells that negatively regulates IFN-I at later time points post-infection. An alternative possibility is that PTPN13 depletion alters VP35 function as the co-factor of the polymerase since VP35 phosphorylation enhances virus replication [68]. We also found that VP35 can interact with PTPN13 reducing VP35 phosphorylation and polymerase activity, which would be in line with enhanced VP35 phosphorylation and enhance replication in PTPN13 KO cells. Therefore, it appears that the effects of PTPN13 can be direct on virus replication as well as indirect by regulating different cytokines, including IFN-I.

We propose that EBOV infection promotes an early, but transient, increase in PTPN13 expression, which results in reduced and controlled production of IFNβ early during infection. As PTPN13 levels decrease over the course of infection, it promotes IFNβ induction. In contrast, in PTPN13 KO cells, there is an early increase in IFNβ induction, presumably as a result of the loss of regulation by PTPN13. As viral replication proceeds, VP35 accumulates, which can then more efficiently inhibit IFNβ and ISGs at later time points.

In addition to decreased ISGs, several cytokines including CXCL1, CXCL3, and CXCL8 also showed decreased levels in PTPN13 KO cells during infection. However, decreased CXCL1 levels also occurred in uninfected PTPN13 knockout cells, implying that PTPN13’s positive regulation of CXCL1 occurs independent of viral infection and impacts basal CXCL1 levels. PTPN13’s positive regulation of PI3K signaling has previously been linked to dephosphorylation of p85β, allowing for it to be ubiquitinated and then degraded to free and activate p110 [42]. Our results corroborate these previous findings while also showing that PTPN13 can serve opposing roles in immune regulation. On one side, PTPN13 is capable of negatively regulating IFNβ while also positively regulating the inflammatory cytokine CXCL1. Together, this suggests that PTPN13 plays an important role in the balance of the immune response, regulating signaling pathways key for inflammation and for viral clearance. Future studies will be needed to determine how PTPN13 controlled immune signaling regulates pathology in vivo. Overall, our results identified a novel role for PTPN13 as a negative regulator of IFNβ production, and indicated that it plays a larger role in balancing the pro- and anti-inflammatory sides of the immune response.

## Data Availability

The RNA sequencing data from Ebola virus-infected cells are available in GEO database with an accession ID: GSE324141.

## Abbreviations

The following abbreviations are used in this manuscript:

**Abbreviation**	**Definition**
BMDCs	Bone Marrow-Derived Dendritic Cells
dsRNA	Double-stranded RNA
EBOV	Ebola virus
EVD	Ebola virus disease
IB	Immunoblot
IFN-I	Type-I Interferon
IP	Immunoprecipitation
IRF3	Interferon regulatory factor 3
KD	Knockdown
KO	Knockout
PACT	Protein activator of PKR
PAMP	Pathogen-associated molecular pattern
PI3K	Phosphoinositide 3-kinase
PRR	Pattern-recognition receptors
PTM	Post-translational modification
PTP	Protein tyrosine phosphatase
PTPN	Protein tyrosine phosphatase nonreceptor type
PTPN13	Protein Tyrosine Phosphatase Non-receptor Type 13
RIG-I	Retinoic acid-inducible gene-I
S	Serine
STAT1	Signal transducer and activator of transcription 1
T	Threonine
TBK1	TANK-binding kinase 1
WCE	Whole cell extract
Y	Tyrosine

## References

[1] Jacob S.T., Crozier I., Fischer W.A., Hewlett A., Kraft C.S., Vega M.-A.D.L., Soka M.J., Wahl V., Griffiths A., Bollinger L. (2020). Ebola Virus Disease. Nat. Rev. Dis. Primers.

[2] Biedenkopf N., Bukreyev A., Chandran K., Di Paola N., Formenty P.B.H., Griffiths A., Hume A.J., Mühlberger E., Netesov S.V., Palacios G. (2023). Renaming of Genera Ebolavirus and Marburgvirus to Orthoebolavirus and Orthomarburgvirus, Respectively, and Introduction of Binomial Species Names within Family Filoviridae. Arch. Virol..

[3] Ebola Virus Disease—Democratic Republic of the Congo. https://www.who.int/emergencies/disease-outbreak-news/item/2025-DON589.

[4] Bixler S., Goff A. (2015). The Role of Cytokines and Chemokines in Filovirus Infection. Viruses.

[5] Basler C.F. (2017). Molecular Pathogenesis of Viral Hemorrhagic Fever. Semin. Immunopathol..

[6] Ficenec S., Bond N., Zifodya J., Schieffelin J. (2025). A Systematic Review of the Immuno-Inflammatory Dysfunction Secondary to Viral Hemorrhagic Fevers; Ebola and Lassa Fever. PLoS Negl. Trop. Dis..

[7] McElroy A.K., Akondy R.S., Davis C.W., Ellebedy A.H., Mehta A.K., Kraft C.S., Lyon G.M., Ribner B.S., Varkey J., Sidney J. (2015). Human Ebola Virus Infection Results in Substantial Immune Activation. Proc. Natl. Acad. Sci. USA.

[8] Basler C.F., Amarasinghe G.K. (2009). Evasion of Interferon Responses by Ebola and Marburg Viruses. J. Interferon Cytokine Res..

[9] Iampietro M., Younan P., Nishida A., Dutta M., Lubaki N.M., Santos R.I., Koup R.A., Katze M.G., Bukreyev A. (2017). Ebola Virus Glycoprotein Directly Triggers T Lymphocyte Death despite of the Lack of Infection. PLoS Pathog..

[10] Iampietro M., Amurri L., Reynard O., Bukreyev A. (2023). Interplay of Ebola Virus With Immune Cells Leading to Their Death by Diverse Mechanisms. J. Infect. Dis..

[11] Reynard S., Journeaux A., Gloaguen E., Schaeffer J., Varet H., Pietrosemoli N., Mateo M., Baillet N., Laouenan C., Raoul H. (2019). Immune Parameters and Outcomes during Ebola Virus Disease. JCI Insight.

[12] Eisfeld A.J., Halfmann P.J., Wendler J.P., Kyle J.E., Burnum-Johnson K.E., Peralta Z., Maemura T., Walters K.B., Watanabe T., Fukuyama S. (2017). Multi-Platform ’Omics Analysis of Human Ebola Virus Disease Pathogenesis. Cell Host Microbe.

[13] Meylan E., Tschopp J., Karin M. (2006). Intracellular Pattern Recognition Receptors in the Host Response. Nature.

[14] Akira S., Uematsu S., Takeuchi O. (2006). Pathogen Recognition and Innate Immunity. Cell.

[15] Swiecki M., Colonna M. (2011). Type I Interferons: Diversity of Sources, Production Pathways and Effects on Immune Responses. Curr. Opin. Virol..

[16] Rehwinkel J., Gack M.U. (2020). RIG-I-like Receptors: Their Regulation and Roles in RNA Sensing. Nat. Rev. Immunol..

[17] Panne D., McWhirter S.M., Maniatis T., Harrison S.C. (2007). Interferon Regulatory Factor 3 Is Regulated by a Dual Phosphorylation-Dependent Switch. J. Biol. Chem..

[18] Mori M., Yoneyama M., Ito T., Takahashi K., Inagaki F., Fujita T. (2004). Identification of Ser-386 of Interferon Regulatory Factor 3 as Critical Target for Inducible Phosphorylation That Determines Activation. J. Biol. Chem..

[19] Luo F., Liu H., Yang S., Fang Y., Zhao Z., Hu Y., Jin Y., Li P., Gao T., Cao C. (2019). Nonreceptor Tyrosine Kinase C-Abl– and Arg-Mediated IRF3 Phosphorylation Regulates Innate Immune Responses by Promoting Type I IFN Production. J. Immunol..

[20] Qin B.Y., Liu C., Srinath H., Lam S.S., Correia J.J., Derynck R., Lin K. (2005). Crystal Structure of IRF-3 in Complex with CBP. Structure.

[21] Thoresen D., Wang W., Galls D., Guo R., Xu L., Pyle A.M. (2021). The Molecular Mechanism of RIG-I Activation and Signaling. Immunol. Rev..

[22] Chiang J.J., Davis M.E., Gack M.U. (2014). Regulation of RIG-I-like Receptor Signaling by Host and Viral Proteins. Cytokine Growth Factor Rev..

[23] Onomoto K., Onoguchi K., Yoneyama M. (2021). Regulation of RIG-I-like Receptor-Mediated Signaling: Interaction between Host and Viral Factors. Cell. Mol. Immunol..

[24] Crosse K.M., Monson E.A., Beard M.R., Helbig K.J. (2018). Interferon-Stimulated Genes as Enhancers of Antiviral Innate Immune Signaling. J. Innate Immun..

[25] Schoggins J.W., Rice C.M. (2011). Interferon-Stimulated Genes and Their Antiviral Effector Functions. Curr. Opin. Virol..

[26] Mühlberger E., Lötfering B., Klenk H.-D., Becker S. (1998). Three of the Four Nucleocapsid Proteins of Marburg Virus, NP, VP35, and L, Are Sufficient To Mediate Replication and Transcription of Marburg Virus-Specific Monocistronic Minigenomes. J. Virol..

[27] Luthra P., Ramanan P., Mire C.E., Weisend C., Tsuda Y., Yen B., Liu G., Leung D.W., Geisbert T.W., Ebihara H. (2013). Mutual Antagonism between the Ebola Virus VP35 Protein and the RIG-I Activator PACT Determines Infection Outcome. Cell Host Microbe.

[28] Prins K.C., Cárdenas W.B., Basler C.F. (2009). Ebola Virus Protein VP35 Impairs the Function of Interferon Regulatory Factor-Activating Kinases IKKε and TBK-1. J. Virol..

[29] Prins K.C., Delpeut S., Leung D.W., Reynard O., Volchkova V.A., Reid S.P., Ramanan P., Cárdenas W.B., Amarasinghe G.K., Volchkov V.E. (2010). Mutations Abrogating VP35 Interaction with Double-Stranded RNA Render Ebola Virus Avirulent in Guinea Pigs. J. Virol..

[30] Woolsey C., Menicucci A.R., Cross R.W., Luthra P., Agans K.N., Borisevich V., Geisbert J.B., Mire C.E., Fenton K.A., Jankeel A. (2019). A VP35 Mutant Ebola Virus Lacks Virulence but Can Elicit Protective Immunity to Wild-Type Virus Challenge. Cell Rep..

[31] Lubaki N.M., Younan P., Santos R.I., Meyer M., Iampietro M., Koup R.A., Bukreyev A. (2016). The Ebola Interferon Inhibiting Domains Attenuate and Dysregulate Cell-Mediated Immune Responses. PLoS Pathog..

[32] Van Tol S., Kalveram B., Ilinykh P.A., Ronk A., Huang K., Aguilera-Aguirre L., Bharaj P., Hage A., Atkins C., Giraldo M.I. (2022). Ubiquitination of Ebola Virus VP35 at Lysine 309 Regulates Viral Transcription and Assembly. PLoS Pathog..

[33] Rodríguez-Salazar C.A., van Tol S., Mailhot O., Gonzalez-Orozco M., Galdino G.T., Warren A.N., Teruel N., Behera P., Afreen K.S., Zhang L. (2024). Ebola Virus VP35 Interacts Non-Covalently with Ubiquitin Chains to Promote Viral Replication. PLoS Biol..

[34] Liu J., Qian C., Cao X. (2016). Post-Translational Modification Control of Innate Immunity. Immunity.

[35] Mustelin T., Vang T., Bottini N. (2005). Protein Tyrosine Phosphatases and the Immune Response. Nat. Rev. Immunol..

[36] Brockdorff J., Williams S., Couture C., Mustelin T. (1999). Dephosphorylation of ZAP-70 and Inhibition of T Cell Activation by Activated SHP1. Eur. J. Immunol..

[37] Sathish J.G., Walters J., Luo J.C., Johnson K.G., LeRoy F.G., Brennan P., Kim K.P., Gygi S.P., Neel B.G., Matthews R.J. (2004). CD22 Is a Functional Ligand for SH2 Domain-Containing Protein-Tyrosine Phosphatase-1 in Primary T Cells. J. Biol. Chem..

[38] Baumgartner C.K., Ebrahimi-Nik H., Iracheta-Vellve A., Hamel K.M., Olander K.E., Davis T.G.R., McGuire K.A., Halvorsen G.T., Avila O.I., Patel C.H. (2023). The PTPN2/PTPN1 Inhibitor ABBV-CLS-484 Unleashes Potent Anti-Tumour Immunity. Nature.

[39] Mcheik S., Aptecar L., Coopman P., D’Hondt V., Freiss G. (2020). Dual Role of the PTPN13 Tyrosine Phosphatase in Cancer. Biomolecules.

[40] Nakai Y., Irie S., Sato T. (2000). Identification of IκBα as a Substrate of Fas-associated Phosphatase-1. Eur. J. Biochem..

[41] Yu H., Lin L., Zhang Z., Zhang H., Hu H. (2020). Targeting NF-κB Pathway for the Therapy of Diseases: Mechanism and Clinical Study. Signal Transduct. Target. Ther..

[42] Kuchay S., Duan S., Schenkein E., Peschiaroli A., Saraf A., Florens L., Washburn M.P., Pagano M. (2013). FBXL2- and PTPL1-Mediated Degradation of P110-Free P85β Regulatory Subunit Controls the PI(3)K Signalling Cascade. Nat. Cell Biol..

[43] Vine V., Scott D.P., Feldmann H., Hoenen T., Groseth A. (2017). Ebolavirus: An Overview of Molecular and Clinical Pathogenesis. Ebolaviruses.

[44] Colavita F., Biava M., Castilletti C., Lanini S., Miccio R., Portella G., Vairo F., Ippolito G., Capobianchi M.R., Di Caro A. (2019). Inflammatory and Humoral Immune Response during Ebola Virus Infection in Survivor and Fatal Cases Occurred in Sierra Leone during the 2014–2016 Outbreak in West Africa. Viruses.

[45] Madaan A., Verma R., Singh A.T., Jain S.K., Jaggi M. (2014). A Stepwise Procedure for Isolation of Murine Bone Marrow and Generation of Dendritic Cells. JBM.

[46] Sanjana N.E., Shalem O., Zhang F. (2014). Improved Vectors and Genome-Wide Libraries for CRISPR Screening. Nat. Methods.

[47] Shalem O., Sanjana N.E., Hartenian E., Shi X., Scott D.A., Mikkelsen T.S., Heckl D., Ebert B.L., Root D.E., Doench J.G. (2014). Genome-Scale CRISPR-Cas9 Knockout Screening in Human Cells. Science.

[48] Kiley M.P., Regnery R.L., Johnson K.M. (1980). Ebola Virus: Identification of Virion Structural Proteins. J. Gen. Virol..

[49] Bradfute S.B. (2023). History and Impact of the Mouse-Adapted Ebola Virus Model. Antivir. Res..

[50] Hoenen T., Watt A., Mora A., Feldmann H. (2014). Modeling the Lifecycle of Ebola Virus under Biosafety Level 2 Conditions with Virus-like Particles Containing Tetracistronic Minigenomes. J. Vis. Exp..

[51] Schneider C.A., Rasband W.S., Eliceiri K.W. (2012). NIH Image to ImageJ: 25 Years of Image Analysis. Nat. Methods.

[52] Bharaj P., Atkins C., Luthra P., Giraldo M.I., Dawes B.E., Miorin L., Johnson J.R., Krogan N.J., Basler C.F., Freiberg A.N. (2017). The Host E3-Ubiquitin Ligase TRIM6 Ubiquitinates the Ebola Virus VP35 Protein and Promotes Virus Replication. J. Virol..

[53] Di Tommaso P., Chatzou M., Floden E.W., Barja P.P., Palumbo E., Notredame C. (2017). Nextflow Enables Reproducible Computational Workflows. Nat. Biotechnol..

[54] Ewels P.A., Peltzer A., Fillinger S., Patel H., Alneberg J., Wilm A., Garcia M.U., Di Tommaso P., Nahnsen S. (2020). The Nf-Core Framework for Community-Curated Bioinformatics Pipelines. Nat. Biotechnol..

[55] Kopylova E., Noé L., Touzet H. (2012). SortMeRNA: Fast and Accurate Filtering of Ribosomal RNAs in Metatranscriptomic Data. Bioinformatics.

[56] Dobin A., Davis C.A., Schlesinger F., Drenkow J., Zaleski C., Jha S., Batut P., Chaisson M., Gingeras T.R. (2013). STAR: Ultrafast Universal RNA-Seq Aligner. Bioinformatics.

[57] Danecek P., Bonfield J.K., Liddle J., Marshall J., Ohan V., Pollard M.O., Whitwham A., Keane T., McCarthy S.A., Davies R.M. (2021). Twelve Years of SAMtools and BCFtools. GigaScience.

[58] Wang L., Wang S., Li W. (2012). RSeQC: Quality Control of RNA-Seq Experiments. Bioinformatics.

[59] García-Alcalde F., Okonechnikov K., Carbonell J., Cruz L.M., Götz S., Tarazona S., Dopazo J., Meyer T.F., Conesa A. (2012). Qualimap: Evaluating next-Generation Sequencing Alignment Data. Bioinformatics.

[60] Okonechnikov K., Conesa A., García-Alcalde F. (2016). Qualimap 2: Advanced Multi-Sample Quality Control for High-Throughput Sequencing Data. Bioinformatics.

[61] Sayols S., Scherzinger D., Klein H. (2016). dupRadar: A Bioconductor Package for the Assessment of PCR Artifacts in RNA-Seq Data. BMC Bioinform..

[62] Daley T., Smith A.D. (2013). Predicting the Molecular Complexity of Sequencing Libraries. Nat. Methods.

[63] Patro R., Duggal G., Love M.I., Irizarry R.A., Kingsford C. (2017). Salmon Provides Fast and Bias-Aware Quantification of Transcript Expression. Nat. Methods.

[64] Love M.I., Huber W., Anders S. (2014). Moderated Estimation of Fold Change and Dispersion for RNA-Seq Data with DESeq2. Genome Biol..

[65] Dauletbaev N., Cammisano M., Herscovitch K., Lands L.C. (2015). Stimulation of the RIG-I/MAVS Pathway by Polyinosinic:Polycytidylic Acid Upregulates IFN-β in Airway Epithelial Cells with Minimal Costimulation of IL-8. J. Immunol..

[66] Escalante C.R., Nistal-Villán E., Shen L., García-Sastre A., Aggarwal A.K. (2007). Structure of IRF-3 Bound to the PRDIII-I Regulatory Element of the Human Interferon-β Enhancer. Mol. Cell.

[67] Ströher U., West E., Bugany H., Klenk H.-D., Schnittler H.-J., Feldmann H. (2001). Infection and Activation of Monocytes by Marburg and Ebola Viruses. J. Virol..

[68] Zhu L., Gao T., Yang W., Liu Y., Liu X., Hu Y., Jin Y., Li P., Xu K., Zou G. (2020). Ebola Virus Replication Is Regulated by the Phosphorylation of Viral Protein VP35. Biochem. Biophys. Res. Commun..

[69] Galão R.P., Wilson H., Schierhorn K.L., Debeljak F., Bodmer B.S., Goldhill D., Hoenen T., Wilson S.J., Swanson C.M., Neil S.J.D. (2022). TRIM25 and ZAP Target the Ebola Virus Ribonucleoprotein Complex to Mediate Interferon-Induced Restriction. PLoS Pathog..

[70] Lo H., Lai T., Li C., Wu W. (2014). TNF-α Induces CXCL1 Chemokine Expression and Release in Human Vascular Endothelial Cells in Vitro via Two Distinct Signaling Pathways. Acta Pharmacol. Sin..

[71] Reid S.P., Valmas C., Martinez O., Sanchez F.M., Basler C.F. (2007). Ebola Virus VP24 Proteins Inhibit the Interaction of NPI-1 Subfamily Karyopherin α Proteins with Activated STAT1. J. Virol..

